# mTOR Signaling and Neural Stem Cells: The Tuberous Sclerosis Complex Model

**DOI:** 10.3390/ijms19051474

**Published:** 2018-05-16

**Authors:** Alice Polchi, Alessandro Magini, Danila Di Meo, Brunella Tancini, Carla Emiliani

**Affiliations:** 1 Department of Chemistry, Biology and Biotechnology, University of Perugia, Via del Giochetto, 06122 Perugia, Italy; alicepolchi@virgilio.it (A.P.); alessandro.magini@unipg.it (A.M.); danila.dimeo@gmail.com (D.D.M.); carla.emiliani@unipg.it (C.E.); 2 Institute for Molecular Cell Biology, University of Münster, Schlossplatz 5, 48149 Münster, Germany; 3 Cells-in-Motion Cluster of Excellence, University of Münster, 48149 Münster, Germany

**Keywords:** mTOR signaling, neural stem cells, tuberous sclerosis complex

## Abstract

The mechanistic target of rapamycin (mTOR), a serine-threonine kinase, plays a pivotal role in regulating cell growth and proliferation. Notably, a great deal of evidence indicates that mTOR signaling is also crucial in controlling proliferation and differentiation of several stem cell compartments. Consequently, dysregulation of the mTOR pathway is often associated with a variety of disease, such as cancer and metabolic and genetic disorders. For instance, hyperactivation of mTORC1 in neural stem cells (NSCs) is associated with the insurgence of neurological manifestation characterizing tuberous sclerosis complex (TSC). In this review, we survey the recent contributions of TSC physiopathology studies to understand the role of mTOR signaling in both neurogenesis and tumorigenesis and discuss how these new insights can contribute to developing new therapeutic strategies for neurological diseases and cancer.

## 1. Introduction 

The mechanistic target of rapamycin (mTOR) is a serine/threonine protein kinase belonging to the phosphoinositide 3-kinase (PI3K)-related kinase family. In mammalian cells, mTOR constitutes the catalytic subunit of two functionally distinct protein complexes, mTOR Complex 1 (mTORC1), which is sensitive to the allosteric inhibitor rapamycin, and mTOR Complex 2 (mTORC2) which, instead, is resistant to acute rapamycin treatment. mTORC1 plays a pivotal role in regulating cell growth and metabolism, while mTORC2 controls cell survival and proliferation and cytoskeletal organization responding to growth signals [1]. In order to promote cell growth, mTORC1 regulates protein synthesis, ribosomal biogenesis and lipogenesis positively while inhibits autophagy [1,2]. Consistent with its central role in the maintenance of cellular homeostasis, mTOR function requires the integration and coordination of multiple intra- and extracellular signals to maintain a balance between anabolic and catabolic processes. mTORC1 activation depends on the cell energy status and is induced by various factors such as nutrient availability, growth factors and energy level through the insulin receptor/phosphoinositide 3-kinase(PI3K)/Akt, the extracellular signal-regulated kinases (ERKs)/ribosomal S6 kinase (RSK) and the AMP-dependent protein kinase (AMPK) signaling pathways. These pathways converge leading to the inhibition by phosphorylation of the Tuberous Sclerosis Complex (TSC), which is a key negative regulator of mTORC1. Once activated, mTORC1 promotes the metabolic processes needed to elicit cell growth [1,3]. Thus, TSC represents the final inhibitor of the mTORC1 signaling pathway and plays a critical role as tumor suppressor. TSC is an heterotrimeric complex made up of hamartin (TSC1), tuberin (TSC2) and the TBC1 domain family member 7 (TBC1D7) [4]. Loss of function mutations in Tuberous Sclerosis Complex 1 (*TSC1*) and *TSC2* genes results in mTORC1 hyperactivation and TSC development. TSC is a monogenic autosomal dominant disease characterized by benign tumors in multiple organs, including brain, kidney and skin, and neurological disorders such as epilepsy, autism and learning impairment [5]. As the molecular bases of TSC lie in the hyperactivation of mTORC1, the symptoms of the disease reflect mTORC1 functions and clearly indicate a role of this complex not only in cellular growth processes, but also in many neurological processes [3,6,7]. During recent decades, our understanding of the role of mTORC1 in neurogenesis and its implication on TSC neurological manifestations has greatly improved thanks to the use of TSC-deficient cell lines and animal models which represent useful tools to provide insights into mTOR neurobiology. In this review, we focus on the current understanding of the role played by mTORC1 in either tumorigenesis and the neurological manifestations of TSC. Moreover, we discuss how the identification of novel component of the TSC1/2-mTORC1 signaling axis can contribute to improve therapies for not only TSC, but also other disorders linked to the dysregulated mTORC1 function. 

## 2. The mTOR Complexes and Their Signaling Network

### 2.1. Structure and Function of mTOR Complexes

**Figure 1:**
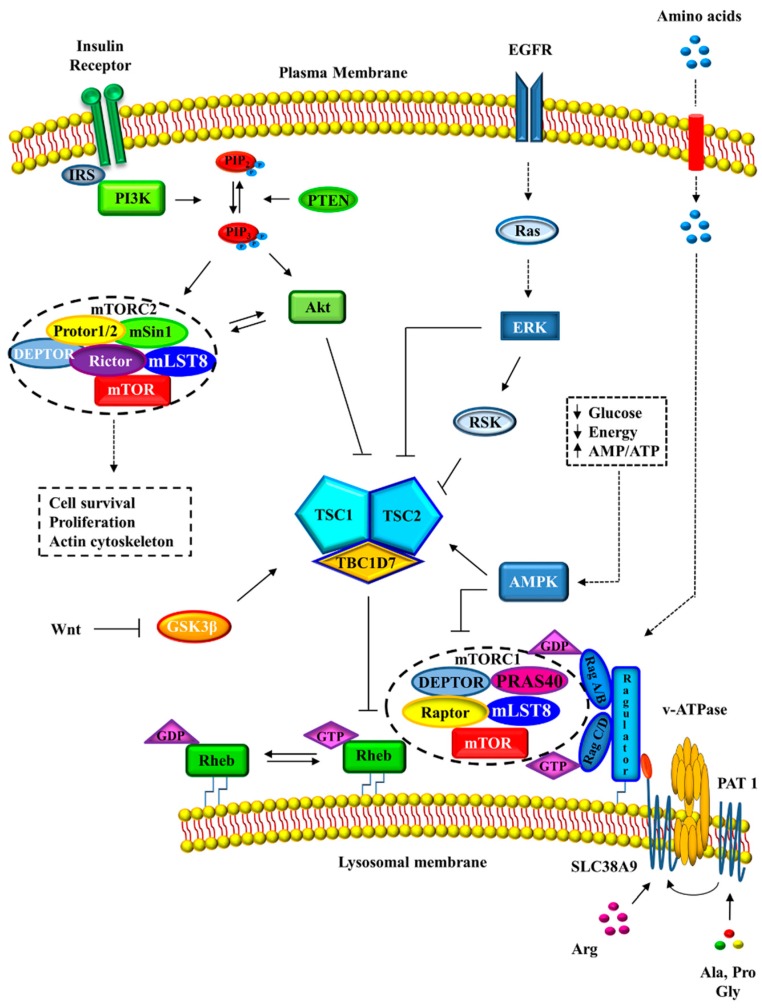
Regulation of mTORC1 activity. mTORC1 and mTORC2 are under the control of numerous upstream signaling pathways that respond to the presence of growth factors, hormones, nutrient availability and stress signals. DEPTOR: DEP domain containing mTOR-interacting protein; EGFR: epidermal growth factor receptor; GSK3β: glycogen synthase kinase 3 beta; IRS: insulin receptor substrate; mLST8: mammalian lethal with Sec13 protein 8; PAT1: proton-coupled amino acid transporter 1; PIP_2_: phosphatidylinositol 4,5-bisphosphate; PIP_3_: phosphatidylinositol 3,4,5-bisphosphate; PRAS40: proline-rich Akt substrate of 40 kDa; PTEN: phosphatase and tensin homolog; Rag: Ras-related GTPases; Raptor: regulatory-associated protein of mTOR; Rheb: Ras homolog enriched in brain; Rictor: rapamycin-insensitive companion of mammalian target of rapamycin; SLC38A9: Solute Carrier Family 38 Member 9; v-ATPase: Vacuolar-type H^+^-ATPase; Wtn: Wingless-type MMTV integration site family.

mTOR is a phosphoinositide 3-kinase related protein kinase (PIKK) with a central role in cell growth and metabolism. The kinase activity of mTOR is closely regulated in response to environmental cues and physiological conditions (Figure 1).

Consistent with its pivotal role on controlling cell function, mTOR deregulation is often associated with the onset of diseases such as neurodegeneration, cancer and diabetes [8,9]. mTOR sequence consists of several conserved structural domains. The region at N-terminal contains multiple repeats called HEAT (for Huntington, EF3, A subunit of PP2A, TOR1), repeats which are involved in protein-protein interactions [10]. The central region and the C-terminus of mTOR contain the FAT (FRAP, ATM, TRAP) and FATC domains which are conserved in other PIKK family members [10]. The FATC region is necessary for mTOR activity. The kinase domain is situated at the C-terminal half, immediately downstream of the FKBP-rapamycin binding (FRB) domain which can interact with the FKBP12-rapamycin complex, inhibiting mTOR activity [11]. mTOR is the catalytic subunit of two functionally and biochemically distinct multiprotein complexes called mTORC1 and mTORC2 [12,13,14]. While mTORC1 plays a central role in cell growth and metabolism regulation, mTORC2 controls cell survival and proliferation as well as cytoskeletal organization responding to growth signals [1]. The significant difference between the two complexes is the diverse sensitivity to rapamycin because mTORC2 is insensitive to acute rapamycin treatment [15]. mTORC1 is a high molecular weight protein complex consisting of five components in which the catalytic subunit, mTOR, is associated with regulatory proteins. The positive regulation of the complex is under the control of two proteins, the regulatory-associated protein of mTOR (Raptor) and the mammalian lethal with Sec13 protein 8 (mLST8 or GβL). In particular, Raptor functions as a scaffold protein and its interaction with mTOR is required for recruitment of specific substrates, [1,10] such as ribosomal S6 kinase (S6K) and eIF4E-binding protein 1 (4E-BP1), through binding to the TOR signaling (TOS) motif [16,17,18]. mLST8 interacts with the catalytic domain, probably by stabilizing the kinase activation, and is essential for mTORC1 activity [1].

In contrast, the proline-rich Akt substrate of 40 kDa (PRAS40) and DEP domain containing mTOR-interacting protein (DEPTOR) act as negative regulators of mTORC1 activity by inhibiting the binding of the substrates [18,19]. The active form of mTORC1 is a dimeric complex of about 1-MDa whose assembly is regulated by the telomere maintenance 2 (TELO2) and TELO2 interacting protein (TTI1/TTI2), that form a TTT complex and interact with mTORC1 during assembly. TTT complex constitutes a larger complex in association with other proteins to control the energy-dependent assembly of functional mTORC1, its dimerization, and its association with Rag for lysosomal localization [20,21].

mTORC1 acts as a sensor in response to energy and nutritional status of the cell to promote cell growth. In particular, in response to nutrients, growth factors, energy and also extracellular stimuli, mTORC1 controls the synthesis of proteins, lipids, and nucleotides, autophagy and both ribosomal and lysosome biogenesis by phosphorylating specific downstream substrates [1,22]. Protein synthesis is promoted by recruitment of the translational machinery through the phosphorylation of S6K and 4E-BP1. Lipid synthesis, necessary for new cell membrane formation, is positively regulated by mTORC1 through the sterol regulatory element binding proteins (SREBP) and the peroxisome proliferator-activated receptor γ (PPARγ), two transcription factors which promote the expression of gene coding for proteins related to lipid and cholesterol homeostasis [19,23]. mTORC1 also plays a key role in the regulation of autophagy. In presence of nutrients, mTORC1 controls, in a negative manner, the autophagic pathway by inhibiting both the activation of Unc-51-like kinase 1 (ULK1), which is necessary in early steps of autophagosome biogenesis, and the nuclear translocation of the transcription factor EB (TFEB), which induces lysosomal and autophagy gene expression [24].

mTORC2 is a complex consisting of six elements including some proteins in common with mTORC1, such as mTOR, mLST8 and DEPTOR, and other different components [19,23]. In mTORC2, the scaffold protein, that assists the complex assembly and the catalytic activity of mTOR, is a rapamycin-insensitive companion of mTOR (Rictor). Additional subunits necessary for the complex activity are the mammalian stress-activated protein kinase interacting protein 1 (mSin1), which acts as a regulatory subunit, and the protein observed with Rictor-1 and Rictor-2 (Protor 1/2) [19,23]. The TTT complex contributes to maintaining stability, as in mTORC1 [23]. mTORC2 is sensitive primarily to growth factors and hormones by promoting actin cytoskeleton organization, cell-cycle entry and cell survival. Moreover, the direct phosphorylation of Akt by mTORC2 enhances cell growth via mTORC1 [25].

### 2.2. mTORC1 Regulation

mTOR functions as a cellular sensor capable of detecting and modulating both intracellular and environmental signals to maintain a homeostatic status. For these reasons, the regulation of mTORC1 activity is under the control of numerous upstream signaling pathways that respond to the presence of growth factors, hormones, nutrient availability and stress signals (Figure 1). The main regulators upstream of mTORC1 are the TSC complex and the Ras homolog enriched in brain (Rheb), a small G-protein which acts as direct activator of mTORC1 [4,26]. The GTPase-activating protein (GAP) activity of TSC2 toward Rheb is controlled by multi-site phosphorylation downstream of several protein kinase signaling pathways among which the PI3K-Akt pathway, the Ras-ERK cascade and AMPK activity [2]. In particular, the direct phosphorylation of TSC2 by Akt promotes the release of Rheb from the TSC complex and activates mTORC1 [27,28]. Similarly, ERK and RSK phosphorylate and inhibit TSC2, promoting mTORC1 activation [29,30]. On the contrary, TSC2 phosphorylation, carried out by AMPK upon energy depletion, increases its GAP activity inactivating mTORC1 [31,32]. Therefore, in the presence of nutrients or growth factors TSC is inhibited and Rheb-GTP can accumulate and activate mTORC1. Otherwise, TSC2 activation leads to GTP hydrolysis by Rheb and the inhibition of mTORC1 function [33].

Importantly, TSC2 phosphorylation status does not influence its GAP activity to Rheb, but its spatial localization. In the absence of growth factor, TSC2 is situated to the lysosomal membranes in proximity of Rheb and mTORC1, while the presence of growth factors promotes its dissociation from the lysosomal membranes [24,27]. In addition, mTORC1 can modulate itself and promote a negative-feedback loop by suppressing both PI3K signaling and mTORC2 activation [19].

Moreover, TCS regulates mTORC1 from the peroxisome in response to reactive oxygen species (ROS) [34]. In this paper, authors demonstrated that peroxisomal ROS stimulate autophagy by activating TSC2 and repressing mTORC1 and suggested that peroxisomal TSC signaling node functions as a cellular sensor for ROS to regulate mTORC1 and autophagy.

Amino acid availability regulates mTORC1 activity independently of growth factor signaling as demonstrated by many studies performed on *Tsc1* or *Tsc2* knockout cells, in which mTORC1 remained sensitive to amino acid deprivation, bypassing TSC complex signaling [35,36,37]. The major proteins modulating mTORC1 activity, with respect to amino acid levels, are the Ras-related GTPases (Rag). The Rag GTPase protein family consists of four members (RagA/B/C/D) in mammals, which assemble in heterodimers (A/B with C/D) at the lysosomal surface [38,39]. Rag GTPase activity depends on their guanine nucleotide state which is regulated by several multi-protein complexes, including the Ragulator and GATOR complexes [40,41]. Although Rag activity does not lead to direct stimulation of mTORC1, Rags are necessary to recruit mTORC1 to the lysosomal membrane where it can interact with Rheb to be activated [38]. It has also been shown that TSC complex is directly involved in the amino acid sensing pathway. In the absence of amino acids, Rags are inactive and can bind and recruit TSC2 to lysosomes. In this manner, Rheb is inhibited causing the release of mTORC1 from the lysosome membrane [42].

Recent studies have shown the existence of Rag GTPase-independent activation of mTORC1 controlled by the presence of glutamine [19]. For example, an in vitro study in RagA/B deficient cells showed how glutamine stimulation caused the translocation to the lysosome of mTORC1 and its activation through a v-ATPase and a small GTPase, adenosine diphosphate ribosylation factor 1 (Arf1) [43].

Cell growth needs nutrients and energy to support the required anabolic processes. Consistent with this, mTORC1 enhances mitochondrial biogenesis to assure the proper energy supply during the cell growth [44]. Moreover, the balance between anabolic and catabolic processes is finely controlled by the energy sensor, AMPK, via multiple mechanisms. In fact, upon energy stress AMPK performs a double negative regulation of mTORC1. Apart of the TSC2 phosphorylation, AMPK inactivates mTORC1 by directly phosphorylating Raptor [24,25].

### 2.3. mTORC1 Signaling Pathway and Cell Growth and Metabolism

**Figure 2:**
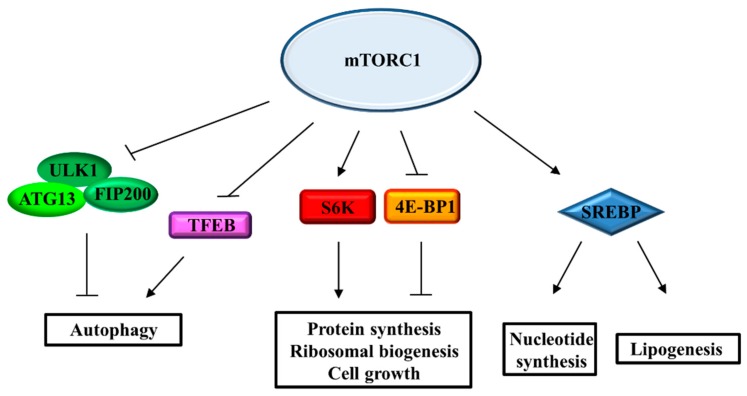
The mTORC1 signaling network driving cell growth. The pathways downstream of mTORC1 signaling controlling protein synthesis, ribosomal biogenesis, metabolism and autophagy. ATG-13: Autophagy-related protein 13; 4E-BP1: eIF4E-binding protein 1; FIP200: focal adhesion kinase family interacting protein of 200 kD; S6K: ribosomal S6 kinases.

mTORC1, considered the hub of many signaling networks, drives cell growth through the regulation of a wide range of anabolic processes, including synthesis of proteins, lipids, and nucleotides (Figure 2). mTORC1 induces protein synthesis in response to cellular growth signals by acting on different transcription and translation effectors [1]. eIF4E-binding protein 1 (4E-BP1) and ribosomal S6 kinases (S6K), which regulate cap-dependent translation initiation and elongation respectively, are direct substrates of mTORC1 activity [45]. Both 4E-BP1 and S6K contain the Tor Signaling Sequence (TOS) motif, necessary to interact with Raptor [16,17,18]. 4E-BP1 acts as negative regulator of cap-dependent protein translation. In the non-phosphorylated state, 4E-BP1 is associated with and inhibits the eukaryotic translation initiation factor 4E (eIF-4E) which, as a consequence, cannot promote the translation of mRNAs involved in cell growth [2]. The phosphorylation by mTORC1 on four Ser/Thr residues of 4E-BP1 induces the release of eIF-4E which can bind to both eIF-4G and eIF-4A and form the active eIF-4F translation initiation complex [46,47]. Also, S6K is a direct effector of mTORC1 and plays an important role in the regulation of mRNA translation and the biogenesis of ribosomes. The mTORC1-dependent activation of S6K leads to the phosphorylation of its own set of targets, many of which promote protein synthesis [48,49]. The primary target of S6K is the ribosomal protein S6, a component of the 40S ribosome subunit which is essential for protein synthesis and cell cycle progression. S6K is also involved in the formation of the eIF-4F translation initiation complex by phosphorylating both eIF-4B, which can be recruited in the complex and increase the eIF4A helicase activity, and the protein programmed cell death 4 (PDCD4), which in its unphosphorylated state acts by inhibiting eIF4A [50]. S6K inhibits the eukaryotic elongation factor 2 kinase (eEF2K), promoting and enhancing translation elongation by eEF2 [51]. Moreover, S6K attends to mRNA maturation by regulating the splicing machinery [52,53]. In support of protein synthesis, mTORC1 directly modulates ribosomal biogenesis by regulating the translation of a subset of mRNAs possessing 5’-terminal oligopyrimidine (TOP) tracts, a class of mRNAs which encodes for essential component of the translational machinery such as ribosomal proteins and both elongation and initiation factors. mTORC1 also promotes rRNAs generation, enhancing the Pol I- and Pol III-dependent transcription [2,25].

To make up for the increasing demand for nucleotides during rRNA production, mTORC1 supports the synthesis of purine and pyrimidine nucleotides. Many studies have demonstrated that mTORC1 signaling induces de novo pyrimidine and, more recently, also purine synthesis. In particular, S6K phosphorylates and activates carbamoyl-phosphate synthetase 2, aspartate transcarbamylase, and dihydroorotase (CAD) enzyme which catalyses the first steps of the pyrimidine biosynthesis pathway [54,55]. In a recent study, it was demonstrated that mTORC1 induces purine synthesis by enhancing the translation of activating transcription factor 4 (ATF4), which regulates the gene-expression of methylenetetrahydrofolate dehydrogenase 2 (MTHFD2), a metabolic enzyme directly involved in the purine pathway [56].

The stimulation of cell growth is also associated with an increase in de novo lipid synthesis, necessary for the biogenesis of plasma membrane and cell organelles. By controlling S6K, lipin1 and CREB regulated transcription coactivator 2 (CRTC2), mTORC1 stimulates lipogenesis trough the activation of the sterol regulatory element-binding proteins (SREBPs), a family of transcription factors that induce the gene-expression of the lipogenic enzymes involved in fatty acids and sterols synthesis [2,57]. Although the involvement of mTORC1 has been demonstrated, the mechanism underlying this regulation has not yet been fully clarified. Moreover, SREBPs, by activating the pentose phosphate pathway, increase the production of NADPH, which is necessary for several biosynthetic processes [58].

## 3. mTORC1 Signaling Pathway and Disease: The Tuberous Sclerosis Complex Model

### 3.1. Clinical Manifestation and Molecular Biology of Tuberous Sclerosis Complex 

Consistent with the central role of mTORC1 in controlling a wide range of cellular functions, the deregulation of its signaling pathways has been implicated in a variety of disorders such as tumorigenesis, epilepsy, cognitive disability, metabolic impairments, neurodegenerative diseases and immunity. TSC represents a good model to study the role of mTORC1 signaling in both tumorigenesis and neurological disorders for its well-defined genetic origin and clinical manifestations.

TSC is a multisystem and autosomal dominant disorder characterized by the development of benign tumors, called hamartomas, in multiple organs such as brain, heart, kidneys, skin and lungs. TSC is consider as a rare disease with an estimated incidence at birth of approximately 1 in 6000 [5]. More than 90% of TSC patients show the presence of three main types of brain lesions: cortical or subcortical tubers, subependymal nodules (SENs) and subependymal giant cell astrocytomas (SEGAs). The brain lesions in TSC are associated with neurological symptoms, such as epilepsy, intellectual disability and autism spectrum disorders (ASDs) [59]. In addition, some patients may show additional neuropsychiatric problems such as anxiety, depression, attention-deficit/hyperactivity disorder (ADHD) and aggressive/disruptive behavior [60]. TSC is caused by inactivating mutations in either *TSC1* [61], or *TSC2* [62] genes. Mutations in *TSC1* or *TSC2* genes were found in about 80–85% of diagnosed TSC cases. In particular, over 200 mutations have been reported in *TSC1* gene and over 700 in *TSC2*, but identification of additional mutations in these genes of the remaining cases is expected thanks to sequencing technology improvements. Although *TSC2* mutations seem to be associated with a more severe phenotype, an obvious genotype-phenotype association is made difficult by the high number of mutations identified [7,63]. Recently, mutations in the *TBC1D7* gene have also been reported, but patients did not present clinical manifestations of TSC [64,65]. Loss of TSC1/TSC2/TBC1D7 complex activity is related to mTORC1 hyperactivation, which characterizes TSC patients’ tissues, inducing several abnormalities in numerous cell biochemical processes such as the activation of transcription, translation and inhibition of autophagy. In accordance with this, numerous studies have revealed upregulation of mTORC1 in cellular and animal models of TSC and strongly indicated mTORC1 activation as the molecular bases of TSC pathology [66,67,68,69]. It is worth noting that the degree of mTORC1 activation correlates well with the severity of pathological symptom [70]. Results obtained using mTOR inhibitors further support the existence of a direct association between hyperactivation of mTORC1 and TSC pathology [71,72]. However, evidence revealing mTORC1-independent functions of TSC1/2, such as the role of TSC2 as transcription factor, has been also produced [73,74,75,76,77]. Further investigation in this regard may be helpful to understand those aspects of TSC that are not unequivocally attributable to mTORC1 activity. A genomic analysis of TSC molecular neuropathology aimed at gaining more insight into the disease at the molecular level has been recently reported [78]. The authors performed RNA sequencing and ribosome-profiling analysis, using a human *TSC2* deleted neural stem cell model, to detect the genome-wide consequences of *TSC2* loss at both the transcriptional and translational levels. Transcriptome analysis of *TSC2*-deficient cells revealed increased expression of genes associated with inflammation processes, indicating an active inflammatory response, in agreement with TSC brain tissue findings. The inflammation state accounts for epileptogenesis and the generation of seizures typically observed in TSC patients. On the other hand, ribosome profiling analysis of the *TSC2* cell model showed elevated protein synthesis in general and a specific increase in the production of angiogenic growth factors, consistent with the high degree of vascularization observed on the tumor lesions of TSC patients [79]. It is worth noting that Grabole et al.′s study demonstrated that mTOR inhibitors corrected translational dysfunction probably implicated in brain tumor hypertrophy but neither inflammation nor angiogenesis associated with transcriptional defects, suggesting the possibility of combining additional treatments for TSC patients based on their specific clinical manifestation [78].

### 3.2. mTORC1 Signalling and Cancer

As previously discussed, mTORC1 controls cell growth and metabolism by positively regulating many related cellular processes such as protein synthesis, ribosome biogenesis, transcription, lipogenesis and nutrient uptake. mTORC1 function is under the control of both oncogenic signaling cascades and cosuppressor effectors, whose mutations can lead to hyperactivation of mTORC1, and, in turn, promote anabolic processes inducing tumor cell growth and proliferation [3]. Among the numerous pathways controlled by mTORC1, the initiation of mRNA translation promoted by the activation of S6K and the promotion of 5′-cap-dependent mRNA translation, induced by the inhibition of the translational repressor 4E-BP1 are supposed to be the most critical for tumor development [80]. According to this, a ribosomal profiling study delineating the translational landscape of prostatic cancer genome revealed a group of mTORC1 translating mRNAs involved in cell proliferation, metabolism and invasion [81].

The constitutive activation of mTORC1 in TSC results in the development of hamartomas in multiple organs. The benign feature of TSC-associated tumors has been, at least in part, attributed to the loss of Akt signaling due to either a mTORC1-dependent negative feedback regulation of Akt and the attenuation of mTORC2 signaling derived from the loss of TSC1/2 complex function [82,83]. More recently, other studies demonstrating that hyperactivated mTORC1-dependent Akt inhibition constrains tumor growth in TSC patients by downregulation of different signaling cascades, have been reported [84,85]. Jin et al. also reported a hyperactivated mTORC1-independent protective effect against malignant tumor development in TSC induced by downregulation of the SOX9-OPN signaling cascade through the inhibition of Akt [86]. Furthermore, Zordan et al. speculated about Akt inhibition, which might also be responsible for the failure of TSC mouse models to reproduce well-defined SENs and full-blown SEGAs. Consistent with this, by inducible conditional transgenesis authors have demonstrated that codeletion of *Tsc1* and *Pten* in postnatal neural stem cells is necessary for the development of SENs and SEGAs closely resembling human TSC lesions highlighting the relevance of both Akt and mTORC2 pathways for the induction of these tumors [87].

The involvement of miRNA in mTORC1-induced tumorigenesis is beginning to emerge. In particular, an increased expression of miR21, miR146a, and miR155 in TSC brain lesions has been reported [88]. Based on these data, Hilaire et al. performed in vitro and in vivo studies to investigate the role of miR-21 in TSC [89]. The authors found that miR-21 is increased approximately 10-fold in *Tsc2*-deficient cells and its inhibition resulted in both reduced tumorigenic potential and increased sensitivity to apoptosis of TSC cells. Interestingly, miR-21 inhibition showed even more striking efficacy when combined with rapamycin. Overall, these insights may contribute to identifying new potential pharmacological targets to develop more effective therapeutic strategies as discussed below.

Apart from the TSC disorder, TSC1/2 loss has also been found in different form of sporadic cancers such as bladder cancer, hepatocellular carcinoma and pancreatic neuroendocrine tumor [90,91,92,93]. This observation suggests that for those form of cancer, characterized by TSC 1/2 loss, it may be possible set up targeted treatment strategies based on the knowledge so far acquired on TSC molecular pathology.

More in general, genetic events, causing aberrant activation of mTORC1 pathways, may be able to induce tumorigenesis through the stimulation of anabolic processes. Consistent with this, gain-of-function mutations on oncogenes as well as loss-of-function mutations in tumor suppressors, which function as up-stream effectors of mTORC1 have been found in a wide variety of human cancers [9,94].

### 3.3. mTORC1 Signalling and Neurogenesis 

mTOR plays key roles in brain physiology and pathology as suggested by the numerous neurological disorders associated with mTOR pathway dysfunction, such as epilepsy, autism and neurodegenerative diseases. In accordance with this, many studies have demonstrated that mTOR is implicated in numerous neurological processes such as neuronal development, circuit formation, synaptic plasticity, learning, memory and feeding [95,96]. In the past, studies on the molecular mechanisms underlying mTOR functions during neurogenesis were hampered because mTOR *null* mice die around embryonic day 6 [97]. The recent development of conditional transgenic mice, that allow affecting mTOR activity during distinct developmental stages, has led to substantial progress in our understanding of the molecular neurobiology of mTOR. In particular, the involvement of mTORC1 in different steps of neurogenesis, as well as in mature neuron functions, has clearly emerged. Cloetta et al. used conditional knockout mice carrying a floxed allele of *raptor* with Nestin-cre to specifically inactivate mTORC1 in the progenitors of the developing CNS. These mice showed a microcephaly starting at E17.5, decreased cell size and reduced cell number, increased cell death and short postnatal survival [98]. Moreover, neurospheres derived from *raptor*-deficient brains displayed defective differentiation of neural progenitors into glia, whereas neuronal differentiation was not affected [98]. To elucidate the role of mTORC1 on neonatal neural stem cell (NSC) self-renewal, Hartman et al. specifically inactivated mTORC1 in NSCs of the subventricular zone (SVZ) of neonatal *Rheb* and *Raptor* knockdown mice [99]. The authors found that mTORC1 inhibition resulted in decreased NSC differentiation and reduced the number of newborn neurons as a consequence of impaired generation of intermediate progenitors, which give rise to neuroblasts. In addition, increasing mTORC1 activity induced terminal NSC differentiation into highly proliferative intermediate progenitors [99]. Interestingly, the inhibition of the translational repressor 4E-BP2, an mTORC1 downstream effector, supported the effect of mTORC1 hyperactivation. On the contrary, mTORC1-activated S6K regulated NSC soma size but not differentiation [99]. Consistent with these findings, Magri et al. using *Tsc1* or *Tsc2* conditional knockout mice showed that activation of mTORC1 in postnatal NSCs reduced their self-renewal and induced premature differentiation, precluding neuronal and oligodendroglial cell maturation [66]. More recently, Mahoney et al., using *Tsc1* floxed transgenic mice investigated whether the effect of hyperactive mTORC1 would be recapitulated in both slow-cycling and quiescent NSCs in the SVZ of young adult mice [100]. Conditional mTORC1 activation resulted in increased production of transit amplifying cells and newborn neuroblasts in the SVZ by inducing differentiation of quiescent NSCs into proliferative daughter cells. In accordance with this, mTORC1 activation did not induce proliferation in slow-cycling NSCs and could be responsible for the progressive loss of NSCs over time [100]. In another study, the effects of *Tsc2* knockout, in developing forebrain excitatory neurons, were evaluated in overall brain development of mutant mice. Mutant mice exhibited distinct neuroanatomical abnormalities, including neuronal hypertrophy and cortical astrogliosis [67]. Altogether, these finding indicate that mTORC1 is critically involved in the regulation of both embryonic and postnatal neurogenesis and is crucial for normal brain development and growth. Moreover, the role of mTORC1 signaling is to drive NSCs differentiation rather than induce proliferation.

Some evidence indicates that mTORC1 also plays a critical role in proper neuron migration. Studies using both *Tsc1* conditional knockout mice and mice constitutively expressing activated *Rheb* proved that mTORC1 pathway hyperactivation in NSCs of the SVZ caused failure of neural progenitor migration to the olfactory bulb and abnormal neuron formation, as observed in TSC neuropathology [66,101,102,103]. Migration defects associated with hyperactive mTORC1 were not only observed in the SVZ. Fu et al. reported that GABAergic interneuron-specific *Tsc1* conditional knockout mice showed impaired growth, decreased survival and displayed ectopic clusters of cells, suggesting impaired interneuron migration [104]. In addition, knockdown of RTP801, an inhibitor of mTOR activation via the TSC complex, strongly affected migration of rat newborn neurons to the cortical plate and resulted in the ectopic localization of mature neurons [105]. Kassai et al. established transgenic mice lines carrying constitutively activated mTOR kinase to selectively direct activation of the mTORC1 pathway in a spatially and temporally controlled manner [106]. The authors found that mTORC1 activation in postmitotic neurons resulted in dysregulation of neuronal cell size and impaired cortical neuronal migration; these abnormalities were mostly attributable to specific effects resulting from mTORC1 signaling activation. The authors also demonstrated that mTORC1 signaling displayed different stage-specific functions in developmental and adult neurons suggesting the involvement of different downstream effectors driving cellular processes in embryonic and adult neurons [106]. Although the effect of mTORC1 loss in neuron migration has been poorly investigated, preliminary studies seem to suggest that mTOR inactivation does not affect neuron migration [107,108]. Thus, while mTORC1 pathway activation strongly perturbs neuronal migration, its inhibition seems to have no consequences. Further investigation about the controversial effect of mTORC1 regulation are needed to clarify the molecular mechanism underling neuronal migration.

### 3.4. mTORC1 Signaling and Neurological Disorders 

TSC is characterized by a high occurrence of epileptic seizures (about 90% of patients) and ASDs (about 50%). This observation has led to associating mTORC1 hyperactivation with the onset of these clinical manifestations. Consistent with this, mice, in which *Tsc1* or *Tsc2* have been deleted in specific neural populations, show hyperactivation of mTORC1, severe epileptic seizures and abnormal social interaction [109,110,111,112]. Abs et al., by inducing acute biallelic *Tsc1* deletion in adult mice, demonstrated a direct role of mTORC1 signaling in epilepsy development in adult brain, also in the absence of a major brain pathology [113]. Zou et al. have recently analyzed the age-dependent pathologic effects of *Tsc1* loss on astrocytes and neurons and evaluated epilepsy development by using an inducible *Tsc1* knock-out mouse. The authors reported that the phenotype observed, by inactivating *Tsc1* at 2 weeks of age, was much less severe compared to prenatal *Tsc1* inactivation, indicating that the severity of neural cell abnormalities and the resulting epilepsy are dependent on the developmental timing of TSC1 loss [114]. Conditional *Pten* knockout mice also exhibit seizures and abnormal social behavior associated with changes in mTOR pathway activity [115,116,117]. Noteworthy, seizures caused by *Tsc1*, *Tsc2* or *Pten* deletion are prevented by rapamycin treatment, indicating that mTOR hyperactivation is a causal link to their occurrence. On the other hand, several studies have reported an increase of mTORC1 activity in chemical convulsant animal models of epilepsy, further supporting the existence of a close association between mTORC1 pathway activation and epileptogenesis [118,119,120]. 

Evidence of the involvement of mTORC1 hyperactivation in autism has also been reported. For instance, the occurrence of an autistic-like behavior was observed using mouse models with specific deletion of *Tsc1* or *Tsc2* in cerebellar Purkinje cells [121,122]. These studies clearly indicated the involvement of mTORC1 signaling in cerebellum function and suggested that dysfunction of Purkinje cells may represent not only a link between TSC and ASD but also a more general neurophysiologic feature that contributes to the ASD phenotype [121]. Moreover, mice with defective translational regulation due to the deletion of *Eif4ebp2*, which encodes for 4E-BP2 an eIF4E repressor downstream of mTOR, exhibited an autistic-like phenotype. [123]. The relevance of the role of mTORC1-dependent protein synthesis in neurophysiological function is clearly emerging and mTORC1-mediated translational deregulation is believed to represent a major cause of mental retardation and autism [7,124].

The strong evidence of a causal link between mTORC1 hyperactivation and both epilepsy and ASD observed in TSC has stimulated researchers to explore mTORC1 pathway activation in other neurological disorders characterized by these clinical manifestations. These studies have led the identification of several syndromes showing altered mTORC1 function. In this regard, the correlation between different forms of autism and mTORC1 signaling dysfunction have been recently discussed by Magdalon et al., who analyzed the mTORC1 pathway in six monogenic ASD-related syndromes as well as in nonsyndromic and idiopathic autism spectrum disorders [124]. On the other hand, it has been recently documented that epilepsy and intellectual disability are clinical manifestations characteristic of phenotypically different disorders associated with cortical developmental malformations secondary to mTOR signaling dysregulation [125].

Moreover, mTORC1 also plays a key role in regulating autophagy, whose dysfunction is involved in the pathogenesis of several neurodegenerative disorders such as Alzheimer′s disease and Parkinson’s’ disease, both characterized by the accumulation of protein aggregates. Autophagy is inhibited by mTORC1 and it has been extensively documented the beneficial effect of autophagy induction by mTOR inhibition in many cellular and animal models of neurodegenerative disorders [126,127,128,129]. As a matter of fact, growing evidence indicates that up-regulated mTORC1 activity is a pathogenic feature common to many neurological disorders, ranging from abnormal brain development to neurodegenerative diseases [6,7]. The existence of a common mechanism underlying mTORC1 hyperactivation and different types of neurological disorders has elicited a great interest because of the potential therapeutic use of mTOR inhibitors and the possible identification of new pharmacological targets for treating these debilitating pathologies [130,131]. On the other hand, inhibition of TOR activity [132,133] and low levels of mTOR in mouse models [134] extends lifespan [135]. The intervention of the mTOR signaling pathway by precise application of small molecular inhibitors will benefit both lifespan and neurological disorders.

## 4. Therapeutic Strategies for Tuberous Sclerosis Complex

### 4.1. Rapalogs: The First Generation of mTOR Inhibitors

Rapamycin was discovered in 1964 by a Canadian expedition to the Island of Rapa Nui. Scientists isolated rapamycin from a bacterial strain of *Streptomyces hygroscopicus* present in soil samples and described it as an antibiotic and antifungal agent [136]. Successively, it was demonstrated that rapamycin showed potent immunosuppressant proprieties, which led to its approval as a drug for preventing allograf rejection [137]. Rapamycin (sirolimus) inhibits mTORC1 by forming an inhibitory complex with the peptidyl-prolyl isomerase FKB506-binding protein (FKP12), which binds to the FRB domain of mTORC1, partially occluding its kinase active site [11]. As consequence, mTORC1 activity is not fully inhibited by rapamycin, which affects mainly S6K (T389) phosphorylation, and has only a limited effect on 4E-BP1 phosphorylation [138]. It has been reported that because 4E-BP1 is one of the critical substrate through which mTORC1 controls cell proliferation, the inefficacy of rapamycin to inhibit its phosphorylation likely contributes to its reduced efficiency as an anti-hyperplastic agent [139]. 

Due to the presence of Rictor, rapamycin-FKB12 complex does not bind to mTORC2, which remains insensitive to acute rapamycin drug administration. However, even if the rapamycin-FKB12 complex does not bind directly to mTORC2, it can bind to mTOR when rapamycin treatment is prolonged. In this way, mTORC2 is inhibited due to the impossibility of the mTOR-rapamycin complex to assemble itself to form new mTORC2 complexes [140].

Activation of the mTOR signaling pathway is the principal causes of TSC and for this reason molecules able to reduce mTORC1 activity have been studied in TSC patients since 2006. Franz and colleagues demonstrated that oral administration of rapamycin caused regression of SEGAs in TSC [141]. However, because of the pharmacological properties of rapamycin, new rapamycin analogs, called rapalogs, with different physicochemical proprieties, have been developed [142]. These are the first generation of mTOR inhibitors, which share a central macrolide structure that is modified at the C-40 position by adding a functional group in order to improve aqueous solubility and oral administration. Among these, everolimus, the hydroxyethyl ester of rapamycin, is a FDA-approved drug for the treatment of different TSC-related morbidities such as SEGAs [143], kidney angiomyolipomas and lymphangioleiomyomatosis [144,145]. Moreover, it has been demonstrated that everolimus reduces the severity of epilepsy and ameliorates neuropsychiatric problems in TSC patients [146,147]. The use of mTOR inhibitors can also be extended for several rare neurodevelopmental disorders in which the activation of the mTORC1 pathway has been verified [131].

Although treatment with mTOR-inhibitors offers significant benefits, their chronic administration is associated with adverse events such as non-infectious pneumonitis, infections, oral ulceration, acne, amenorrhoea, disturbed wound healing, and metabolic events (e.g., hyperglycemia, dyslipidemia) [148,149,150]. Cessation of treatment with mTOR inhibitors is associated with the regrowth of tumors in TSC patients [149].

### 4.2. Novel Therapeutic Approaches for Tuberous Sclerosis Complex

**Figure 3:**
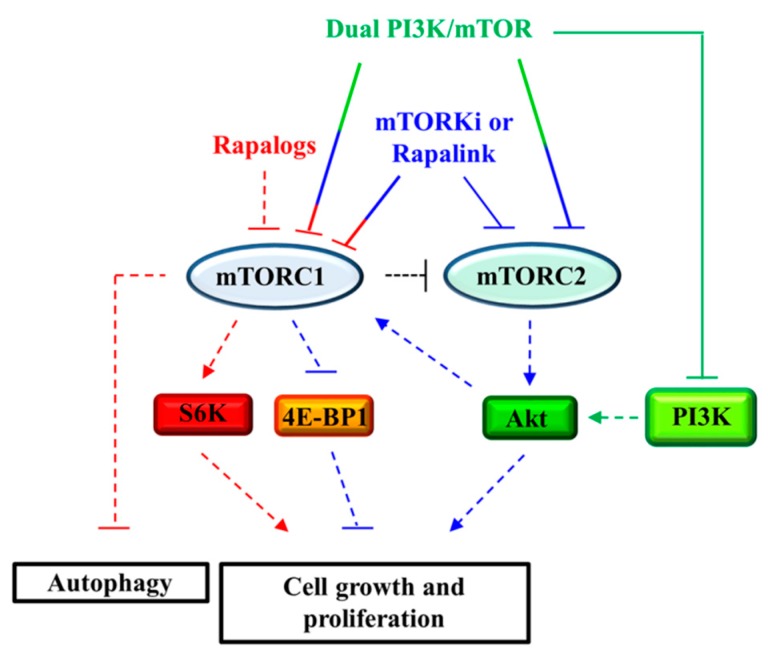
Effects of drugs on mTORC1 and mTORC2 signaling on autophagy and cell proliferation. Pharmacological targets of first generation mTOR inhibitors (rapalogs; red), dual PI3K/mTOR inhibitors (green), second generation mTOR inhibitors (mTORKi; blue), and third generation mTOR inhibitors (Rapalink; blue) have been shown. Dashed/solid line T-bar: inibition; dashed/solid line arrow: activation.

Because of both their limited effects on tumor growth and the systemic effects on the immune system linked to the chronicity of treatments performed using mTOR inhibitors, research for the identification of new therapeutic strategies is moving forward (Figure 3). 

As already noted, the residual activity of mTORC1 on 4E-BP1 protein after rapamycin administration makes it as a cytostatic drug. To overcome this problem the “second generation” mTOR inhibitors, called mTORKi, has been developed [80,151]. These molecules bind to the ATP catalytic domain of mTOR, inhibiting both the mTORC1 ability to phosphorylate both S6K and 4E-BP1, and the mTORC2 activity. This makes these molecules potent inhibitors of cell growth and proliferation, which could be useful for the treatment of specific tumors arising in TSC. In any case, the use of the “second generation” of mTOR inhibitors, as well as rapalogs, removes the negative feedback activity of S6K on PI3K that leads to the re-activation of the PI3K/Akt pathway. In some cases, the administration of an mTOR inhibitor promotes cells survival and inhibit the apoptosis process. In 2008, Tabernero and colleagues demonstrated that everolimus administration caused the activation of Akt in patients with advanced solid tumors [152].

The limited effects of both the first and the second generation of mTOR inhibitors on tumor growth could be overcome by using a combined treatment. With this in mind, rapamycin and MK-2206, an Akt inhibitor, have been tested on *Tsc1*- and *Tsc2*-null MEFs demonstrating a strong cytotoxic effect. The benefit of the combined treatment was also demonstrated in a TSC xenograft mouse model [153]. Wang et al. showed that in both *Tsc1*- and *Tsc2*-null MEFs the hyperactivation of mTORC1 drives Akt inhibition through the downregulation of FOXO3a/PDGFRα pathway [84]. Rapamycin treatment led to the upregulation of PDGFRα that has been associated with the increase in tumorigenesis in *Tsc1*- or *Tsc2*-knockout MEFs. Based on this evidence, the authors proposed to use a combination of rapamycin and AG1295, an inhibitor of PDGFRα, which showed the ability to reduce the growth in cells lacking the TSC complex, both in vitro and in vivo. Another paper showed a reduction in COX2 expression in *Tsc2*-null MEFs mediated by STAT3 signaling. The administration of rapamycin in combination with celecoxib, a COX2 inhibitor, strongly inhibited *Tsc2*-deficient cell growth [85].

Moreover, new compounds, called dual PI3K/mTOR inhibitors has been developed, showing the ability to inactivate both Akt and 4E-BP1 activity. Among these, inhibitors such as NVP-BEZ235 (Novartis) [154], and GSK2126458 (GlaxoSmithKline) can be mentioned [155]. These drugs showed a better anti-tumor activity with respect to the first generation of mTOR inhibitors because they were able to inhibit PI3K and suppress the activation of both mTOR complexes.

The use of mTOR inhibitors, even in cases where they have proved to be very effective, has led to the selection of resistant drug mutations on mTOR, which cause the generation of resistant clones. To overcome these issues, Rodrik-Outmezguine et colleagues [156] generated the “third generation” mTOR inhibitor, the so called Rapalink, which showed strong anti-tumor activity in breast cancer cells expressing the wild-type allele of mTOR or its resistant forms for both rapalogs and mTORKi. Rapalink has been designed to bind both the ATP- and the FRB-binding sites of mTOR. This bivalent interaction allows the inhibition of the mTOR complexes and their rapamycin- and AZD8055-resistant forms in breast cancer cell lines.

One of the most important function of mTORC1 is its control of the autophagy pathway. In nutrient-replete conditions, mTORC1 inhibited the autophagy process by phosphorylating the autophagosomal-driven formation protein ULK1 and TFEB. In some cases, the inhibition of mTORC1 activity, induced by mTOR inhibitors, could be related to the induction of cytoprotective autophagy. In these cases, combination therapies with autophagy inhibitors could be used; these inhibitors are able to potentiate the effects of rapalogs [157]. A combined strategy using rapamycin and resveratrol, a natural autophagy regulator, showed a selective induction of apoptosis in *Tsc2* knockout cells [158,159]. However, the inhibition of autophagy in TSC treatment is controversial. For example, Liang et colleagues [160] suggested that in cells lacking either TSC1 or TSC2 there is an accumulation of YAP protein caused by the impairment of autophagy.

Another promising target for TSC therapy seems to be the cellular defense system against reactive oxygen species [161]. Different papers have shown that the use of Hsp90 inhibitors and/or GSH synthesis inhibitors induces TSC cell death [162]. In a recent paper, it has been demonstrated that p62/sequestosome-1, an autophagic substrate, is necessary to sustain the production of intracellular GSH needed to limit mitochondrial damage and promote the proliferation of *Tsc2* knockout cells [163].

As already reported, there is different evidence revealing mTORC1-independent functions of TSC1/2 [73,74,75,76,77]. This information could be very useful to develop new therapeutic strategies bypassing mTOR signaling pathway avoiding severe adverse events related to mTOR-inhibitor long term treatments. For example, the Bordey group [164] showed that filamin A is overexpressed in *Tsc1*-null neurons and demonstrated that this increase was mTOR independent and associated to the MEK-ERK1/2 pathway. In another work, it has been shown that TSC2 reduction in newborn neurons causes abnormalities in leading processes of migrating neurons. The authors demonstrated that Reelin-Dab1 signaling is dysregulated in TSC mouse models and in cortical tubers from TSC patients causing the increase of the E3 ubiquitin ligase Cul5 expression. Interestingly, the reduction of Cul5 restored normal neuron migration [165]. Recently, we demonstrate that TFEB activation restores *Tsc1*-deficent neural stem/progenitor cell migration. In this work, we suggested TFEB as a druggable protein target for SEGAs therapy that can be used for an mTOR-independent approach [69].

Finally, our group has patented a method for alternative route of administration of mTOR inhibitors directly into the brain with the aim to maintain the beneficial effects of mTOR inhibition and to reduce the side effects of systemic treatment [166].

## 5. Conclusions and Future Directions

mTORC1 signaling pathways play a pivotal role in regulating cell growth through both sensing the energetic status of the cell and coordinating most metabolic processes that underlie growth. Consistent with this, dysregulation of mTORC1 activity is often associated with a variety of disease including those characterized by uncontrolled cell growth such as cancer and those disorders characterized by neurological dysfunctions such as epilepsy and ASDs. In recent decades, extensive studies have led to elucidate new features of mTOR function and regulation and considerable progress in our understanding of the pathological mechanisms associated to mTORC1 hyperactivation has been made. From these findings it is clearly emerging that mTORC1 dysfunctions may be associated not only with prototypical mTOR-opathies such as TSC, but also with some sporadic forms of cancer as well as with different disorders characterized by neuronal development malformations. However, due to the remarkable complexity of the mTOR signaling network and the numerous pathways involved, further studies are needed to fully elucidate the molecular mechanisms underlying mTOR function and the interplay with the other cell and organism functions. It is worth noting that new insights into mTOR molecular biology may contribute to identifying other potential pharmacological targets to set up therapeutic strategies specifically tailored for each particular disorder.
